# A functional methylation signature to predict the prognosis of Chinese lung adenocarcinoma based on TCGA

**DOI:** 10.1002/cam4.4431

**Published:** 2021-12-02

**Authors:** Ke Wang, Ying Liu, Guanzhong Lu, Jinrong Xiao, Jiao Huang, Lin Lei, Ji Peng, Yangkai Li, Sheng Wei

**Affiliations:** ^1^ Medical College Hubei University of Arts and Science Xiangyang Hubei China; ^2^ Department of Epidemiology and Biostatistics Ministry of Education Key Laboratory of Environment and Health School of Public Health Tongji Medical College Huazhong University of Science and Technology Wuhan Hubei China; ^3^ Department of Cancer Control Shenzhen Center for Chronic Disease Control Shenzhen Guangdong China; ^4^ Department of Thoracic Surgery Tongji Hospital Tongji Medical College Huazhong University of Science and Technology Wuhan Hubei China

**Keywords:** biological mechanism, DNA methylation, lung adenocarcinoma, prediction model, prognosis

## Abstract

**Background:**

Lung cancer is the leading cause of cancer morbidity and mortality worldwide, however, the individualized treatment is still unsatisfactory. DNA methylation can affect gene regulation and may be one of the most valuable biomarkers in predicting the prognosis of lung adenocarcinoma. This study was aimed to identify methylation CpG sites that may be used to predict lung adenocarcinoma prognosis.

**Methods:**

The Cancer Genome Atlas (TCGA) database was used to detect methylation CpG sites associated with lung adenocarcinoma prognosis and construct a methylation signature model. Then, a Chinese cohort was carried out to estimate the association between methylation and lung adenocarcinoma prognosis. Biological function studies, including demethylation treatment, cell proliferative capacity, and gene expression changes in lung adenocarcinoma cell lines, were further performed.

**Results:**

In the TCGA set, three methylation CpG sites were selected that were associated with lung adenocarcinoma prognosis (cg14517217, cg15386964, and cg18878992). The risk of mortality was increased in lung adenocarcinoma patients with the gradual increase level of methylation signature based on three methylation sites levels (HR = 45.30, 95% CI = 26.69–66.83; *p* < 0.001). The C‐statistic value increased to 0.77 when age, gender, and other clinical variables were added to the signature to prediction model. A similar situation was confirmed in Chinese lung adenocarcinoma cohort. In the biological function studies, the proliferative capacity of cell lines was inhibited when the cells were demethylated with 5‐aza‐2'‐deoxycytidine (5‐aza‐2dC). The mRNA and protein expression levels of *SEPT9* and *HIST1H2BH* (cg14517217 and cg15386964) were downregulated with different concentrations of 5‐aza‐2dC treatment, while cg18878992 showed the opposite result.

**Conclusion:**

This study is the first to develop a three‐CpG‐based model for lung adenocarcinoma, which is a practical and useful tool for prognostic prediction that has been validated in a Chinese population.

## Graphical abstract

1. This study is the first to employ a three‐CpG‐based model for lung adenocarcinoma.
2. The methylation signature explored in TCGA can be validated in Chinese population.
3. The three‐CpG‐based model can predict the prognosis of lung adenocarcinoma well.

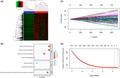

## 1 INTRODUCTION

Lung cancer is the leading cause of cancer morbidity and mortality worldwide, contributing to 13.0% of newly diagnosed cancers and 19.4% of cancer‐related deaths.^1^ Moreover, the percent has risen to 17.1% of new cases and 21.7% of cancer deaths due to lung cancer in China.^2^ As one of the most important pathological types of lung cancer, in recent years, individualized lung adenocarcinoma treatments, including EGFR TKIs and EML4‐ALK, have been highly effective for the treatment of patients with EGFR mutations or harboring EML4‐ALK fusions.^3^, ^4^ Although these recent developments in targeted treatment have been used for lung adenocarcinoma, patients without such mutations generally lack sensitivity to these targeted treatments compared to those with mutations, leading to an unsatisfactory 5‐year survival rate.^5^ Thus, continued studies to improve the efficacy of treatment for lung adenocarcinoma are crucial. Exploring the molecular and epigenetic features of lung adenocarcinoma during tumor occurrence and development may be beneficial for lung adenocarcinoma therapy.

The unsatisfactory nature of individualized therapy reflects the need to elucidate underlying mechanisms other than gene alterations to promote the treatment for lung cancer. There have been studies reported that epigenetic changes are closely associated with lung cancer tumorigenesis besides genetic alteration.^6^, ^7^ As one important mechanism of epigenetic changes, DNA methylation can not only affect chromatin construction, but also closely related to gene expression regulation system.^8^ Abnormal methylation status can lead to disturbance of several genes, and has been shown to correlate with the potential aggressiveness of tumors.^9^

Recent studies have reported that aberrant DNA methylation often happens in early lung cancer.^10^, ^11^, ^12^ In addition, some studies have found a phenomenon of abnormal methylation status at certain CpG sites in some lung cancer tissues.^13^, ^14^, ^15^ Tang et al. found that compared with lung cancer patients with an unmethylated *DAPK* gene promoter, patients with abnormal hypermethylation had a worse prognosis.^16^ Other studies have showed that the levels of methylation CpG sites located on the gene promoter CpG islands in *CHD13*, *RASSF1A*, *and BCAT1* are higher in lung cancer tumor tissue than in adjacent normal tissue.^17^, ^18^ Thus, the methylation level of certain CpG sites may represent potential molecular markers that may have an important effect in prognosis prediction of lung cancer patients. However, considering the lack of genome‐wide DNA methylation analyses with multiple samples, there are only a few studies that have explored which DNA methylation markers are most valuable in predicting the prognosis of lung adenocarcinoma.

In consideration that TCGA database can provide large omics data, this study first analyzed the DNA methylation patterns of lung adenocarcinoma patients, identified differentially methylation CpG sites (DMCs), and then screened for methylation CpG sites associated with lung adenocarcinoma prognosis. A methylation signature was subsequently identified that can be directly incorporated into a clinical test for prognostic prediction. A Chinese lung adenocarcinoma cohort was used to validate the correlation between DNA methylation previously identified in TCGA set and lung adenocarcinoma prognosis. Moreover, the biological function of methylation CpG sites was further performed in cell lines.

## 2 METHODS

### 2.1 TCGA data of lung adenocarcinoma patients

The omics data in TCGA for lung adenocarcinoma were used in this study, including methylation, mRNA expression, and clinical information (https://portal.gdc.cancer.gov/). The information of DNA methylation (level three) was generated using the Illumina Human Methylation 450 platform for all samples on 1 October 2017. The gene expression data (level three) were generated from the Illumina HiSeq RNA‐seq V2 platform. The clinical data were downloaded for survival analysis at the same time. In total, 418 patients for whom both TCGA methylation array and clinical data were available and 26 adjacent normal samples from the TCGA dataset were included in this study.

### 2.2 Screening of methylation CpG sites associated with the prognosis of lung adenocarcinoma

According to TCGA database, 48,2421 CpG probes were collected in the Illumina Human Methylation 450 of lung adenocarcinoma in total. And we removed 8,8621 probes showed “NA” values in more than 25% samples and ultimately obtained 39,3800 CpG probes. The Illumina Human Methylation 450 data of tumor and adjacent normal samples in lung adenocarcinoma were gathered at the same time by the same standards.

DMCs between tumor tissue and adjacent tissue were defined as false discovery rate (FDR) <0.05 for the comparison between tumor tissue methylation level and adjacent tissue methylation level, and the absolute value of log_2_ (fold change) >2.5. Bioinformatics analysis including GO enrichment analysis and cluster analysis were also performed. LASSO Cox regression method was analyzed to confirm methylation CpG sites associated with lung adenocarcinoma prognosis based on DMCs. A methylation signature was built according to the sum of each methylation CpG level times the regression coefficient of LASSO Cox regression.^19^, ^20^

### 2.3 Chinese lung adenocarcinoma cohort for the validation set

To validate the effect of methylation CpG sites found in the TCGA database on Chinese lung adenocarcinoma prognosis, information for people with pathologically confirmed primary lung adenocarcinoma was collected from Wuhan Tongji Hospital from January 2014 to December 2014. A systematic questionnaire survey was performed to collect basic characteristics information of lung adenocarcinoma patients, such as age, gender, history of smoking, and drinking et al. Clinicopathologic information acquisition including cancer stage, degree of tumor differentiation, histology, chemotherapy, and radiation treatment history before operation were from the individual medical records of each patient. The standard of tumor staging was carried out according to the UICC/AJCC in terms of tumor‐node‐metastasis staging system.^21^ The tumor tissues and some adjacent normal tissues of participants were collected.

Patient selection criteria for enrollment are as follows: (1) the diagnosis of histological and pathological for lung adenocarcinoma criteria must meet the criteria of WHO, (2) the patient had no record of having received any other anti‐cancer treatment before surgery, (3) the basic characteristics information and clinicopathologic information is available, and (4) the patients can be kept in touch during follow‐up.

### 2.4 Follow‐up of lung adenocarcinoma patients

All the participants included in this study were followed up every 3 to 4 months in the first 2 years and every 6 to 7 months thereafter. The follow‐up process was mainly conducted by trained and experienced doctors, which included the state of life (alive or dead), the treatment (chemotherapy or radiation therapy) and recurrence and metastasis after operation. Follow‐up information on the therapeutic efficacy and patient prognosis was collected through interviews, telephone calls, and questionnaires. The interval between diagnosis date and death date or the date of last follow‐up was defined as survival time. Participants were followed until December 2017.

### 2.5 DNA isolation and pyrosequencing

Total DNA from tumor samples and adjacent normal samples were isolated referring to the instructions of DNA isolation kit (TIANGEN, Beijing, China). DNA Bisulfite Conversion Kit (QIAGEN, Hilden, Germany) was used for sodium bisulfite treatment of extracted DNA and then pyrosequenced. PyroMark Assay Design 2.0 (QIAGEN, Hilden, Germany) was used to design primers for each methylation CpG site. The experiment conditions of PCR amplification were 40 cycles containing denaturation at 98°C for 10 s, annealing at 55.0°C for 30 s, and extension at 72°C for 30 s, with PyroMark PCR Kit (QIAGEN, Hilden, Germany). Pyrosequencing was used to quantify CpG methylation levels based on the PCR products with manufacturer's instructions of PyroMark Q96 instrument (QIAGEN, Hilden, Germany).

### 2.6 RNA isolation and quantitative RT‐PCR

Total RNA from patient samples was isolated referring to the instructions of TRIzol reagent (Invitrogen, Carlsbad, USA). Primer design of target genes was performed with Primer Premier 5.0. Pre‐designed random primers were used to reverse transcribe total RNA (500 ng) with a volume of 10 µl, referring to PrimeScript RT Reagent Kit instructions (TaKaRa, Dalian, China). SYBR Green Real‐time PCR (RT‐PCR) was applied to measure gene (ABI [Foster, USA] 7500). The mRNA expression level of target gene was calculated with the 2 × 2^−△△Ct^ method. In addition, △Ct was defined as the difference between the level in target gene and reference gene (GAPDH) in threshold cycles, and △△Ct value for one gene was calculated by the difference value between △Ct of sample and △Ct of standard. The specific primer sequences of the target genes and GAPDH are listed in Supplementary materials.

### 2.7 Protein isolation and Western blotting

Samples of lung adenocarcinoma participants were gathered for protein detection. RIPA buffer with protease inhibitor cocktail (Roche, Basel, Switzerland) was used for sample preparation and the supernatants were then resuspended in sample buffer. After denaturation of the samples by the way of boiling for 10 min, 30% polyacrylamide gel was used for protein electrophoresis with the same amount (40 μg). Whereafter, PVDF membrane was used for protein transfer (Millipore, Massachusetts, USA). Blocking buffer was used to block membranes for 2 h, then the following antibodies were used SEPT9 (Abcam [Cambridge, USA] ab38314, 1:500), HIST1H2BH (Abcam ab1790, 1:1000); MAPT (Abcam ab64193, 1:500), GAPDH (Goodhere [Hangzhou, China] AB‐P‐R 001, 1:1000), and finally overnight under the condition of 4°C. The membranes were in a state of HRP‐linked secondary antibodies (Boster [Wuhan, China] BA1054, 1:50,000) for 2 h after cleaned by TBST for three times on the following day. An electrochemiluminescence (ECL) detection kit (Thermo [Waltham, USA] NCI5079) was used for immunoreactive protein bands observation at the completion of washing. Azure c300 Gel Imaging System (Azure Biosystems, Dublin, USA) and BandScan software was used to scan and quantify protein bands. Three times have been done for all of the experiments.

### 2.8 Cell lines

Lung adenocarcinoma cell lines A549 and NCI‐H1975, as well as immortalized human bronchial epithelial cells MRC‐5 were acquired from American Type Culture Collection (ATCC, Manassas, USA), and then identified not longer than 6 months after cell passage by ATCC for this study. A549, NCI‐H1975, and MRC‐5 were maintained in Dulbeccoʼs modified Eagle medium (Invitrogen, Carlsbad, USA) including fetal bovine serum (10%) and penicillin–streptomycin (0.1 mmol/L, 5%) at an environment of 37 °C and 5% CO_2_.

### 2.9 Treatment with 5‐aza‐2dC

Cells were seeded at a density of 1.0×10^4^/ml 24 h before treatment. Cells were demethylated with 5‐aza‐2dC (Sigma‐Aldrich, St Louis, USA) at a concentration gradient of 0, 1, 5, and 10 μmol/L for 6 days. Culture medium containing 5‐aza‐2dC was changed every 24 h. Total RNA was isolated and used for RT‐qPCR. For cell growth assays, the cell lines were then dealt with different concentration gradients of 5‐aza‐2dC, and an MTT assay were performed with three multiple holes.

### 2.10 Statistical analysis

Population characteristics are showed as the mean ± standard deviation (SD) and counts, respectively. In order to compare the differences of quantitative data between groups, Student's *t*‐test, one‐way ANOVA as well as Mann–Whitney *U* test were performed and for qualitative data comparison, Pearson chi‐squared test was analyzed. To assess the association between DNA methylation and lung adenocarcinoma prognosis, unconditional Cox regression method was performed. The methylation signature was analyzed in all lung adenocarcinoma patients of TCGA dataset and Chinese dataset, and the participants were defined as high‐ and low‐risk groups based on a cut off value of median. Grouping analyses were performed to evaluate the association between methylation CpG sites and lung adenocarcinoma risk. The prognostic prediction efficiency of the methylation signature was estimated with Harrell's C‐statistic. The correlation between DNA methylation and gene expression or gene expression level and protein level was analyzed using a linear correlation model.

*p* value less than 0.05 was supposed to be significantly different (two sided). GraphPad Prism 5 software was performed to generate pictures. SAS version 9.4 (SAS Institute, Cary, USA) was used for data analyses.

## 3 RESULTS

### 3.1 The discovery set (TCGA database)

#### 3.1.1 Characteristics of the lung adenocarcinoma patients and screening for methylated CpG sites

**FIGURE 1:**
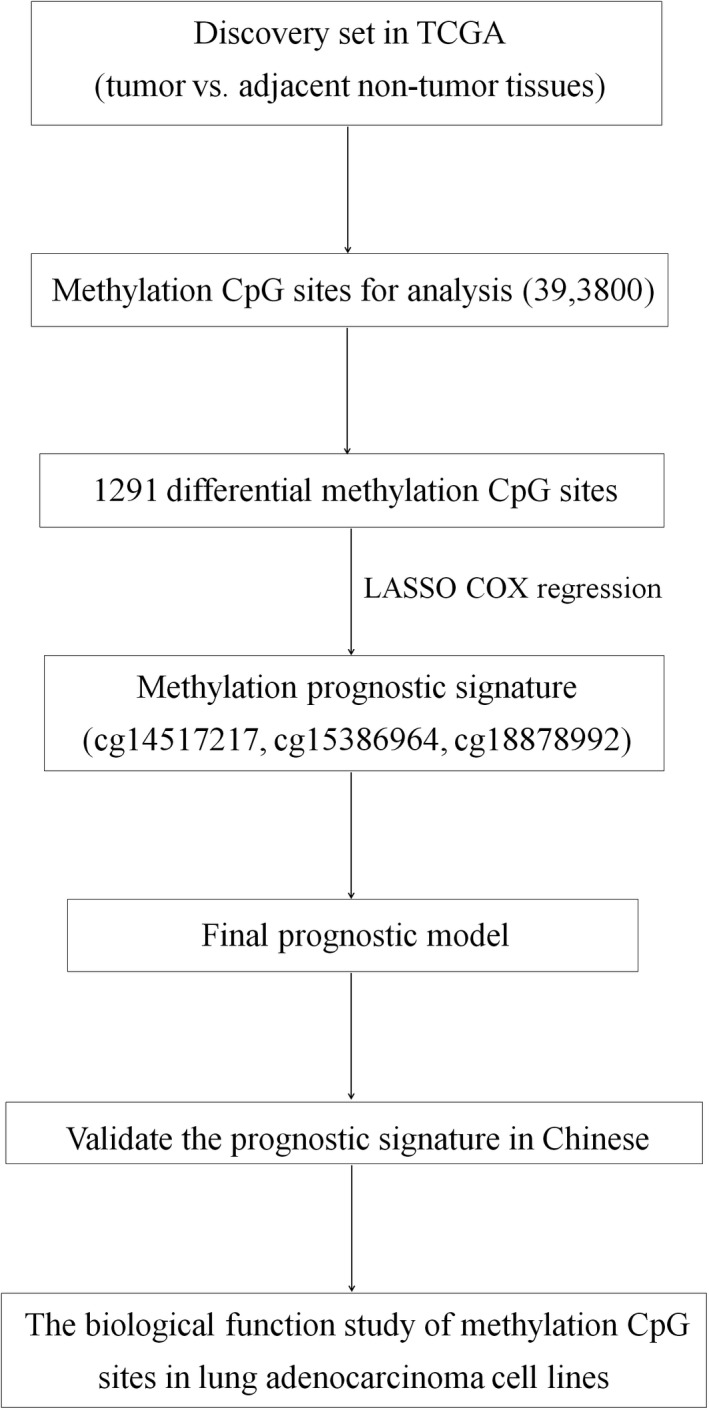
Flow chart of the study design. Twenty‐six paired tumor and adjacent normal samples of lung adenocarcinoma patients were used for candidate methylation CpG islands screening with Illumina Human Methylation 450 platform in TCGA set. Moreover, LASSO Cox regression method was used to build a methylation signature model in the validation set. In addition, the three‐CpG‐based prognostic signature was validated in a Chinese dataset. Biological function study of methylation CpG sites in lung adenocarcinoma cell lines was further performed

The flow chart indicating the study design in the discovery set based on the TCGA set is shown in Figure 1. A total of 418 lung adenocarcinoma patients with information on both TCGA methylation array and clinical data were included. The clinical characteristics of lung adenocarcinoma based on TCGA set are presented in Table 1. As shown, 195 patients were males (46.7%) and the mean age was 65.10 (± 10.14) years. A total of 339 subjects reported a history of smoking (84.8%). During the follow‐up, 102 (24.4%) patients died, and 316 (75.6%) patients were still alive. The mean survival time of all participants was 6.75 (1.60–22.28) months.

**TABLE 1:** Baseline characteristics of lung adenocarcinoma based on TCGA set (*n* = 418)

Characteristic ^a^	Results
Age (years)	65.10 ± 10.14
Gender	
Male	195 (46.7%)
Female	223 (53.3%)
Race	
White	336 (80.4%)
Others	82 (19.6%)
History of smoking	
Yes	339 (84.8%)
No	61 (15.2%)
Clinical stage	
Stage I	227 (55.0%)
Stage II	99 (24.0%)
Stage III	67 (16.2%)
Stage IV	20 (4.8%)
Chemotherapy history	
Yes	152 (36.4%)
No	266 (63.6%)
Radiotherapy history	
Yes	88 (21.1%)
No	330 (78.9%)
Death	
Yes	102 (24.4%)
No	316 (75.6%)
Survival time (month)	6.75 (1.60–22.28)

Abbreviation: TCGA, The Cancer Genome Atlas.

^a^ Characteristics are showed as the mean ± standard deviation or median (quartile1–quartile3) for quantitative data, and number (percentage) for categorical data.

**FIGURE 2:**
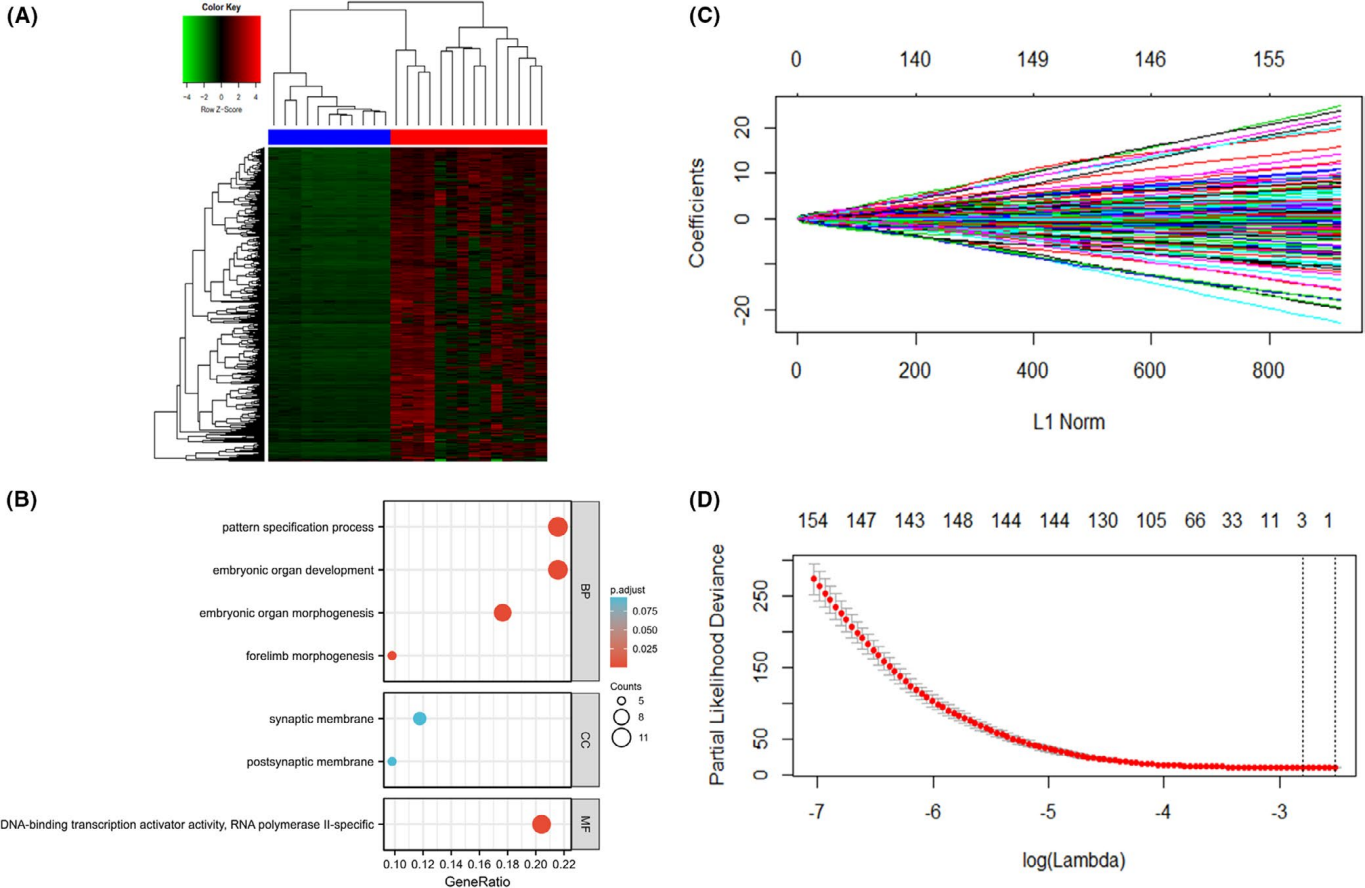
Bioinformatics analysis based on the TCGA set. (A) Heatmap showing the top 100 differentially methylation CpGs in 26 paired tumor and adjacent normal tissues of lung adenocarcinoma. (B) GO enrichment analysis using the top 100 differentially methylation CpGs in 26 paired samples. (C) and (D) LASSO Cox regression analysis based on the TCGA set. (C) LASSO coefficient information of candidate methylation CpG islands. (D) Two dotted vertical lines were drawn in the optimal criteria of 1‐s.e. and with a result of three nonzero coefficients. Three CpGs, cg14517217, cg15386964, and cg18878992, with coefficients of 0.45, 0.04, and −0.17, were eventually incorporated into the model

The screening of methylation CpG sites was based on the TCGA set. A total of 14,9486 methylated CpG sites had an FDR < 0.05 in the comparison between the tumor tissue methylation level and adjacent tissue methylation level. Moreover, 1291 methylation CpG sites defined as DMCs had an absolute value of log_2_ (fold change) greater than 2.5. A heatmap showing the top 100 differentially methylation CpGs in lung adenocarcinoma tumor and adjacent nontumor tissues (26 samples) is presented in Figure 2A. The distributions between hyper‐ and hypomethylated CpG sites appeared to be asymmetric, and the tumor samples tended to have hypermethylated CpG sites. GO enrichment analysis was performed using the top 100 DMCs, and the results of enrichment in biological process (BP), cellular component (CC), and molecular function (MF) are presented in Figure 2B.

#### 3.1.2 Methylation CpG sites associated with lung adenocarcinoma prognosis

LASSO Cox regression method was analyzed to confirm methylation CpG sites associated with lung adenocarcinoma prognosis. Three significant methylation CpG sites were included in the final model: cg14517217, cg15386964, and cg18878992 (Figure 2C,D), the meaningful target gene of each methylation CpG site was *SEPT9*, *HIST1H2BH*, *MAPT*, respectively. The gene annotation information of three methylation CpG islands selected by LASSO Cox regression in the Human Methylation 450 Array of TCGA datasets is shown in Table S1. The regression coefficients of three methylation CpG islands in the optimal LASSO Cox regression model were 0.45, 0.04, and −0.17 (Table S2).

According to the optimal LASSO Cox regression model, a methylation signature for every lung adenocarcinoma participant was built: methylation signature = (0.45 × cg14517217) + (0.04 × cg15386964) − (0.17 × cg18878992). The participants were grouped into high‐risk and low‐risk, based on a cut off value defined as the median value of methylation signature. In TCGA set, the cut off value was 0.02.

The identified CpG islands and methylation signature levels grouped by clinical and pathological characteristics of lung adenocarcinoma patients in TCGA set are shown in Table S3. As shown, compared with the subjects without a history of smoking, the level of methylation signature was significantly higher in smokers (0.06 ± 0.10 vs. 0.02 ± 0.07, *p* = 0.010). The similar trend was shown in objects with different clinical disease stages (0.04 ± 0.09 vs. 0.07 ± 0.11, *p* = 0.007).

#### 3.1.3 Association between methylation signature and lung adenocarcinoma prognosis

Cox regression model was used to analyze the association between methylation signature and lung adenocarcinoma prognosis (Table 2). After adjustment for basic clinical variables, the risk of mortality was increased with the gradual increase level of methylation signature (HR = 45.30, 95% CI = 26.69–66.83; *p* < 0.001). When the subjects were grouped into high‐risk and low‐risk, subjects in high‐risk group showed a 123% higher risk of mortality in comparison with low‐risk subjects (HR = 2.23, 95% CI = 1.41–3.54; *p* < 0.001).

**TABLE 2:** The association between methylation signature level and the prognosis of lung adenocarcinoma in TCGA set and Chinese cohort

Population	Crude HR (95% CI)	Adjusted HR (95% CI)^a^	Adjusted HR (95% CI) ^b^
TCGA set			
Methylation signature	33.80 (20.43–47.54)**	54.90 (38.72–75.60)*	45.30 (26.69, 66.83)**
Subgroup			
Low risk	Ref	Ref	Ref
High risk	2.33 (1.55–3.51)*	2.35 (1.51–3.67)**	2.23 (1.41, 3.54)**
Chinese cohort			
Methylation signature	5.06 (2.08–8.34)**	4.85 (1.84–9.87)*	8.81 (4.72, 18.51)**
Subgroup			
Low risk	Ref	Ref	Ref
High risk	1.26 (1.16–3.87)*	1.50 (1.16–4.91)*	8.52 (5.52, 10.58)*

Note

*p < 0.05; **p < 0.001.

Abbreviations: 95% CI, 95% confidence interval; HR, hazard ratio; TCGA, The Cancer Genome Atlas.

^a^ Adjustment for age, gender, race, and smoking status.

^b^ Adjustment for age, gender, race, history of smoking, chemotherapy history, radiotherapy history, and clinical stage.

In subgroup analysis, the methylation signature showed a more pronounced protective effect on the younger group, females, nonsmokers, and those who had received postoperative chemotherapy (Table 3). We also demonstrated that the association between methylation signature and the prognosis of lung adenocarcinoma had some differences due to the characteristics of tumor. The methylation signature showed a stronger protective effect on early‐stage patients (stage I) than late‐stage patients (stage II/III/IV).

**TABLE 3:** The association between methylation signature and the prognosis of lung adenocarcinoma in TCGA set and Chinese cohort by subgroup

Characteristics	HR (95% CI)^a^	HR (95% CI)^b^
Age (years)^c^		
<65	28.36 (22.17–48.99)*	1.77 (0.87–3.96)
≥65	38.52 (32.60–40.08)**	6.61 (1.85–9.59)*
Gender^d^		
Male	19.02 (15.12–24.18)*	8.01 (5.87–14.33)*
Female	20.53 (11.56–29.71)*	10.99 (3.27–17.51)*
History of smoking^d^		
Yes	49.49 (37.15–62.38)*	57.47 (43.51–71.24)**
No	27.13 (10.02–32.41)*	15.84 (10.08–21.48)*
Clinical stage^d^		
Stage I	49.71 (31.68–67.40)*	4.26 (1.24–7.59)*
Stage II/III/IV	58.58 (35.14–67.90)**	12.64 (10.21–15.88)**
Chemotherapy history^d^		
Yes	10.42 (6.29–17.97)**	1.34 (0.38–4.49)
No	26.07 (22.37–35.00)*	9.46 (3.60–14.87)*

Note

*p < 0.05; **p < 0.001.

Abbreviations: 95% CI, 95% confidence interval; HR, hazard ratio; TCGA, The Cancer Genome Atlas.

^a^ HR (95%CI) in TCGA set.

^b^ HR (95%CI) in Chinese lung adenocarcinoma patients.

^c^ Adjustment for gender, race, history of smoking, chemotherapy history, radiotherapy history, and clinical stage.

^d^ Adjustment for other variables.

#### 3.1.4 The prognostic prediction efficiency of the methylation signature

The prognostic prediction efficiency of the methylation signature was estimated with Harrell's C‐statistic (Table 4). It was shown that the C‐statistic value was 0.66 (95% CI = 0.62–0.69) with the methylation signature alone used to evaluate the prediction efficiency on the prognosis of lung adenocarcinoma. When age, gender, race, and other clinical variables were included to make a prediction model, the C‐statistic value showed a marked increase to 0.77 (95% CI = 0.73–0.81). The C‐statistic value increased by 16.7 percent overall.

**TABLE 4:** The prognostic prediction efficiency of methylation signature and prediction model in TCGA set and Chinese cohort

Population	C‐statistic	95% CI
TCGA set		
Methylation signature	0.66	0.62–0.69
Prediction model^a^	0.77	0.73–0.81
Chinese cohort		
Methylation signature	0.61	0.57–0.68
Prediction model^a^	0.73	0.70–0.79

Abbreviations: 95% CI, 95% confidence interval; TCGA, The Cancer Genome Atlas.

^a^ Prediction model was built based on methylation signature and some clinical variables (age, gender, race, history of smoking, chemotherapy history, radiotherapy history, and clinical stage).

In addition, we analyzed the correlation between DNA methylation and target gene expression (Figure S1). mRNA levels of *SEPT9* and *HIST1H2BH* in tumor tissues were significantly higher than those in adjacent normal tissues, while that of *MAPT* was lower (*p* < 0.05). The correlation coefficients between DNA methylation and gene expression of these three methylation CpG sites were 0.32, 0.71, and −0.35, respectively (all *p* values < 0.001).

### 3.2 The validation set (Chinese lung adenocarcinoma cohort)

#### 3.2.1 Characteristics of lung adenocarcinoma patients in the Chinese population

One hundred and sixteen Chinese participants diagnosed with lung adenocarcinoma were incorporated into validation study set. One hundred and sixteen tumor tissues and 30 adjacent normal tissues were collected and used in the subsequent study. The basic characteristics of lung adenocarcinoma patients are presented in Table S4. As shown, 56 patients were males (48.3%) with a mean (± SD) age of 60.27 (± 8.57) years. Forty‐five subjects reported a history of smoking (38.8%). During the follow‐up, 20 (17.2%) participants died, and 96 (82.8%) participants were still alive. The mean survival time of all participants was 24.18 (16.71–27.00) months.

#### 3.2.2 The level of methylation CpG sites associated with lung adenocarcinoma prognosis

Pyrosequencing was used to quantify the CpG site methylation levels associated with lung adenocarcinoma prognosis found in TCGA set. The sequences of all primers are shown in Table S5. The methylation levels of cg14517217, cg15386964, and cg18878992 were detected using pyrosequencing in 116 tumor tissues and 30 adjacent normal tissues (Figure S2). Compared with adjacent normal tissue, DNA methylation levels of three CpG sites were differently higher in tumor tissue (0.09 ± 0.12 vs. 0.01 ± 0.00, *p* < 0.001 for cg14517217; 0.15 ± 0.13 vs. 0.03 ± 0.01, *p* < 0.001 for cg15386964 and 0.08 ± 0.09 vs. 0.03 ± 0.01, *p* = 0.005 for cg18878992, respectively), as shown in Figure S3 (left panel).

The identified CpG islands and methylation signature levels grouped by the basic characteristics of Chinese lung adenocarcinoma are shown in Table S6. As shown, compared with the subjects without a history of smoking, the level of methylation signature was significantly higher in smokers (0.15 ± 0.46 vs. −0.02 ± 0.28, *p* = 0.013). The similar trend was also shown for patients in different age groups and clinical stages.

#### 3.2.3 Association between methylation signature and lung adenocarcinoma prognosis

The association between methylation signature and lung adenocarcinoma prognosis in Chinese cohort is shown in Table 2. After adjustment for basic clinical variables, the prognosis of Chinese patients worsened with increasing methylation signature levels (HR = 8.81, 95% CI = 4.72–18.51; *p* < 0.001). When subjects were grouped into high‐risk and low‐risk, subjects in high‐risk group had a 7.52 times higher risk of death than low‐risk subjects (HR = 8.52, 95% CI = 5.52–10.58; *p* = 0.002).

We also performed a stratified analysis in the validation set, as shown in Table 3. Similar to the results in the discovery set, the methylation signature showed a more pronounced protective effect on the younger group, nonsmokers, and those who had received postoperative chemotherapy. Moreover, the association between methylation signature and the prognosis of lung adenocarcinoma had some differences due to the characteristics of tumor. The methylation signature showed a stronger protective effect on early‐stage patients (stage I) than late‐stage patients (stage II/III/IV).

#### 3.2.4 The prognostic prediction efficiency of the methylation signature

The prognostic prediction efficiency of the methylation signature was also estimated in Chinese lung adenocarcinoma cohort (Table 4). It was shown that the C‐statistic value was 0.61 (95% CI = 0.57–0.68) when only methylation signature was used to evaluate the prediction efficiency on lung adenocarcinoma prognosis. When age, gender, and other clinical variables were included in the prediction model, the C‐statistic value showed a marked increase to 0.73 (95% CI = 0.70–0.79). The C‐statistic increased by 19.7 percent overall.

In addition, RT‐PCR as well as Western blotting were conducted to measure the level of gene expression and protein expression of target genes (*SEPT9*, *HIST1H2BH*, and *MAPT*). The specific primer sequences of the target genes and GAPDH are listed in Table S7. In the analyses to validate the correlation between DNA methylation and gene expression as well as gene expression and protein expression, it was found that mRNA levels of *SEPT9* and *HIST1H2BH* in tumor tissues were significantly higher than those in adjacent normal tissue, while that of *MAPT* was lower (*p* < 0.05). The correlation coefficients between DNA methylation and gene expression of these three methylated CpG sites were 0.31 for cg14517217, 0.41 for cg15386964, and −0.28 for cg18878992. The same result was seen in the correlation between gene expression and protein expression (data not shown). Furthermore, we have found that the mRNA levels of *SEPT9* and *HIST1H2BH* were higher in tumor tissue in comparison with adjacent tissue in 30 lung adenocarcinoma patients and lower in *MAPT* (Figure S3, right panel).

### 3.3 Biological function studies

#### 3.3.1 The influence of 5‐aza‐2dC on the methylation level

**FIGURE 3:**
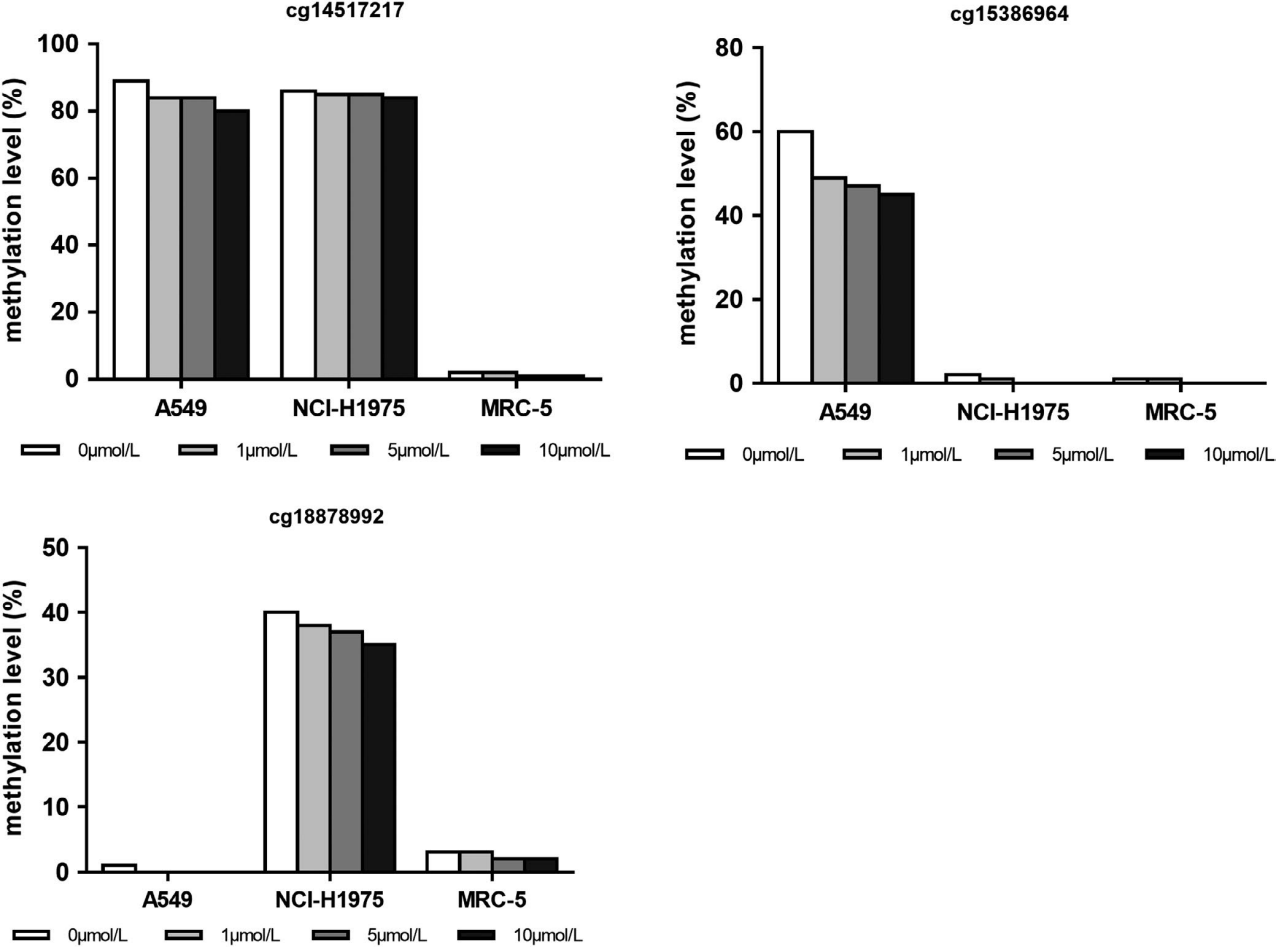
The influence of 5‐aza‐2dC on the methylation level in different cell lines. Human lung adenocarcinoma A549 cells, NCI‐H1975 cells, and immortalized human bronchial epithelia MRC‐5 cells were cultured in vitro. Different concentrations of 5‐aza‐2dC were added to these three cell lines: 0 μmol/L (control group), 1 μmol/L (low‐demethylated group), 5 μmol/L (middle‐demethylated group), and 10 μmol/L (high‐demethylated group). Pyrosequencing was conducted to validate the CpG methylation levels

Human lung adenocarcinoma A549 cells, NCI‐H1975 cells, and immortalized human bronchial epithelia MRC‐5 cells were cultured in vitro. Different concentrations of 5‐aza‐2dC were added to these three cell lines: 0 μmol/L (control group), 1 μmol/L (low‐demethylated group), 5 μmol/L (middle‐demethylated group), and 10 μmol/L (high‐demethylated group). Pyrosequencing was conducted to validate CpG methylation levels in cell lines. As shown in Figure 3, in the control groups, the MRC‐5 cell line was slightly methylated with low methylation levels of cg14517217, cg15386964, and cg18878992, while the A549 cell line was highly methylated, with high methylation levels of cg14517217 and cg15386964. The NCI‐H1975 cell line was also highly methylated, with high methylation levels of cg14517217 and cg18878992. Without treatment, cg18878992 was slightly methylated in A549 and the methylation level of cg15386964 was slightly methylated in NCI‐H1975. Thus, the following study focused on cg14517217 in A549, NCI‐H1975, and MRC‐5, cg15386964 in A549 and MRC‐5, and cg18878992 in NCI‐H1975 and MRC‐5. The effect of demethylation via 5‐aza‐2dC treatment was not obvious in MRC‐5, while in A549 and NCI‐H1975, the methylation level of the three methylated CpG sites decreased with the increasing concentration of 5‐aza‐2dC.

#### 3.3.2 The proliferative capacity of cells after 5‐aza‐2dC treatment

**FIGURE 4:**
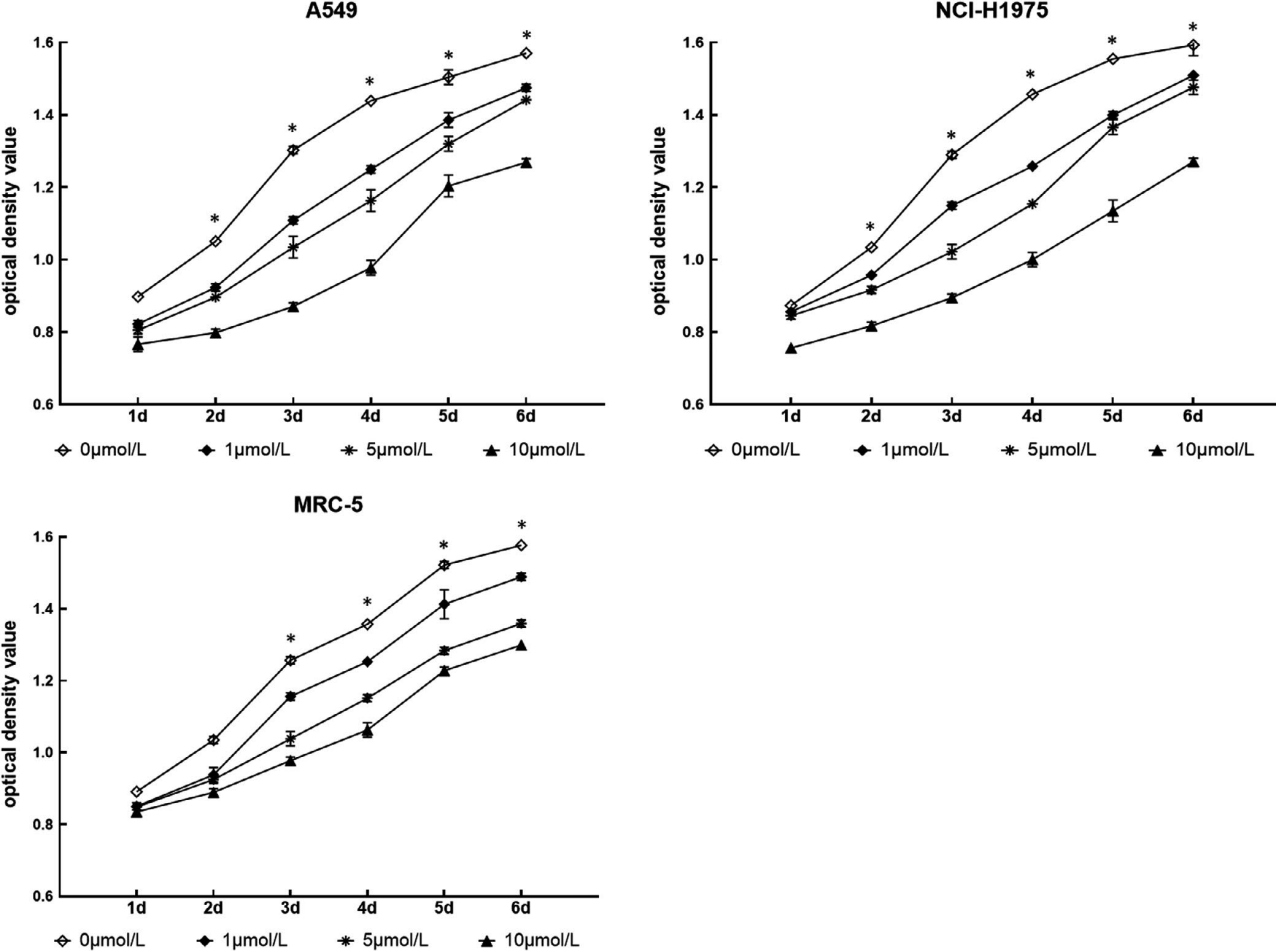
Cell growth analysis via the MTT assay. A549, NCI‐H1975, and MRC‐5 cells were demethylated with 5‐aza‐2dC at different concentrations (0 μmol/L, 1 μmol/L, 5 μmol/L, and 10 μmol/L). MTT was performed to evaluate cell growth for 6 days. *p < 0.05

MTT was performed to detect the proliferative capacity after dealing with 5‐aza‐2dC in different concentration gradients. The proliferative capacity of cell lines was inhibited when the cells were demethylated with 5‐aza‐2dC, especially in A549 and NCI‐H1975 cells (Figure 4). The proliferation rate of cell lines significantly decreased with the concentration of 10 μmol/L compared with 5 μmol/L, 1 μmol/L 5‐aza‐2dC treatment and control group after 6 days.

#### 3.3.3 Gene expression and protein expression of target genes with 5‐aza‐2dC treatment

**FIGURE 5:**
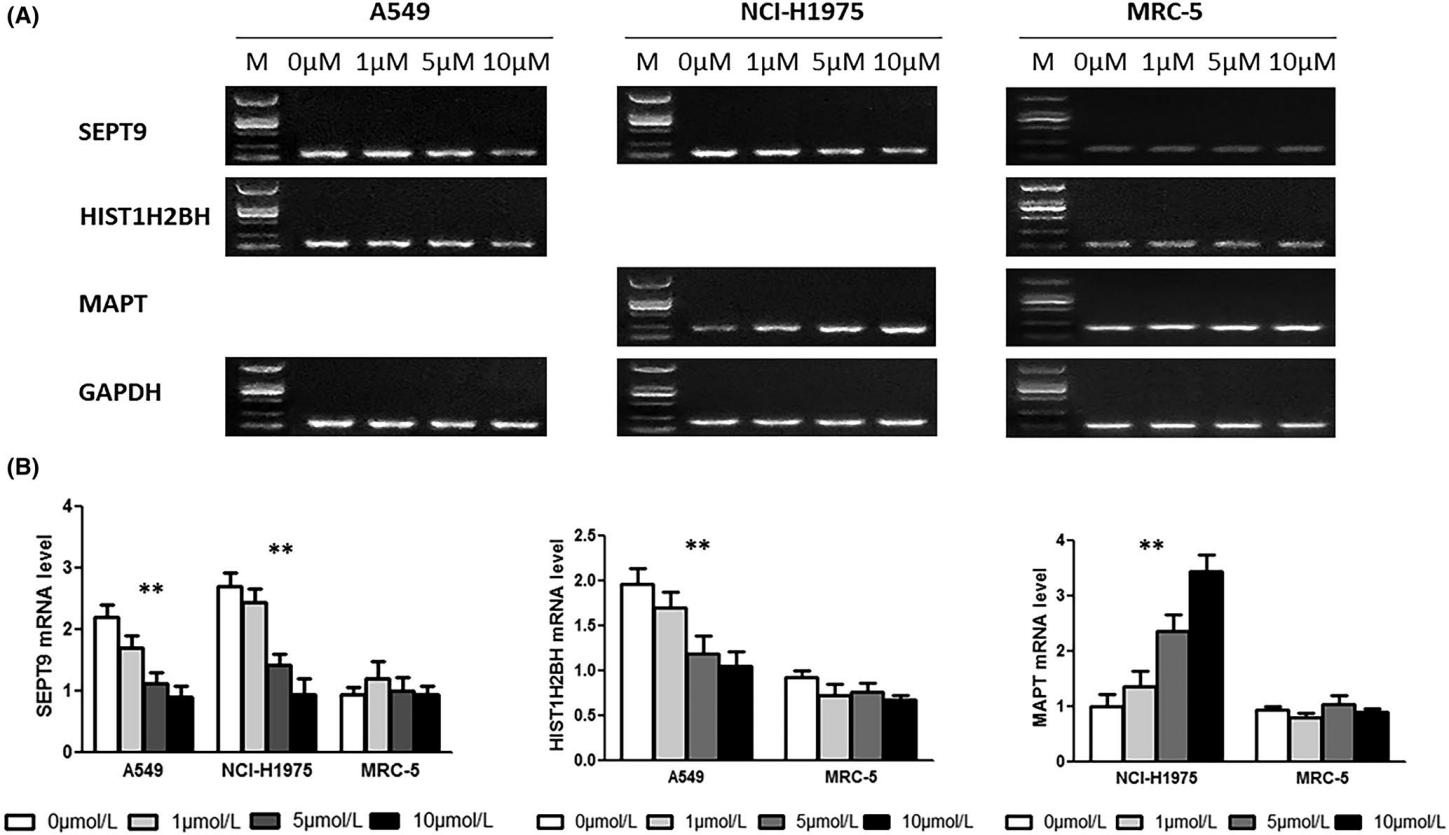
The gene expression of three methylation CpG islands in three cell lines by RT‐PCR. (A) RT‐PCR showing the gene expression of three methylation CpG islands in cells demethylated with 5‐aza‐2dC at different concentrations. (B) The gene expression of three methylation CpG islands in cells demethylated with 5‐aza‐2dC. **p < 0.001

RT‐PCR as well as western blotting were conducted to measure the gene and protein levels in cell lines. As shown, 5‐Aza‐2dC treatment had no obvious effect on mRNA level or protein expression level in the MRC‐5 cell line. In A549 and NCI‐H1975 cells, mRNA and protein levels of *SEPT9* and *HIST1H2BH* (cg14517217 and cg15386964) were downregulated with a concentration gradient of 5‐aza‐2dC treatment, while the mRNA and protein levels of *MAPT* (cg18878992) were upregulated (Figure 5 and Figure S4).

## 4 DISCUSSION

In this study, we first found three methylation CpG sites which were associated with lung adenocarcinoma prognosis (cg14517217, cg15386964, and cg18878992) based on the TCGA database. The association between methylation signature and lung adenocarcinoma prognosis was explored, and the outcome of multivariate Cox regression model revealed that the risk of mortality increased with the gradual increase level of methylation signature in lung adenocarcinoma patients. Moreover, the prognostic prediction model based on methylation signature showed a satisfactory efficiency for lung adenocarcinoma. Interestingly, DNA methylation of these three CpG sites was significantly correlated with target gene expression. In validation set, the same conclusion was obtained and confirmed in a Chinese lung adenocarcinoma cohort. Exploration of the biological mechanism underlying the methylation of these CpG sites in human lung adenocarcinoma cell lines revealed that changes in the methylation level could affect mRNA and protein expression of target gene. The biological mechanism of methylation in the development of lung adenocarcinoma may occur through the causal relationship between DNA methylation and target gene/protein expression.

Building a model by combining multiple biomarkers may significantly improve the predictive ability in comparison with single biomarker.^22^ Recently, few genome‐wide studies have focused on the DNA methylation model in lung adenocarcinoma.^23^, ^24^, ^25^ Wang et al. have found a nine‑gene methylation signature for lung adenocarcinoma prognosis with the data from TCGA and Gene Expression Omnibus (GEO).^23^ Cai et al. have defined a methylation signature including 16 CpG sites for lung adenocarcinoma prognosis with the data from TCGA. Cox regression method was used to confirm methylation CpG sites associated with the prognosis of lung adenocarcinoma.^24^ Wang Y et al. have built a methylation signature including 16 CpG sites for lung adenocarcinoma prognosis with the data from TCGA, regardless of the difference between tumor tissue and adjacent tissue as well as the important clinical characteristics, and with a predict value of 0.68 for the prediction model.^25^ However, no population validation and functional experiments were performed in these studies. Moreover, the methods of statistics for sequencing data analysis in some early researches may not suitable. As the most commonly used statistical method for survival analysis, Cox regression method is not applicable when analyzing high‐dimensional sequencing data.^26^, ^27^ In this study, LASSO Cox model was performed as an effective way to solve this problem.^28^, ^29^

In our study, a three‐CpG‐based model was built and can efficiently predict the prognosis of lung adenocarcinoma based on TCGA set. Given the three biomarkers, our model may be more acceptable and more likely to be applied in clinical practice in comparison with other studies. The prediction model in this study has a kind of ability to evaluate the prognosis of lung adenocarcinoma patients accurately. We further performed a validation study in a Chinese cohort, and the prognostic accuracy of the prediction model was found to be still effective. Therefore, the prediction model may have potential prognostic value and is feasible for use in the clinic.

This study is the first to report three methylation sites (cg14517217, cg15386964, and cg18878992) associated with lung adenocarcinoma prognosis in Chinese set. cg14517217 is located in the CpG island sequence on chromosome 17 in the gene body region of the *SEPT9* (septin9) gene. As a protein‐coding gene, *SEPT9* is a member of the membrane protein family and encodes a GTP‐binding protein. Moreover, *SEPT9* is associated with the regulation of cell division and the cell cycle and has significant effect on tumor occurrence and development as a tumor oncogene or suppressor gene.^30^ Recent studies have found abnormal methylation status of *SEPT9* promoter in several cancers and this status can affect the condition of prognosis, especially for colorectal cancer patients.^31^, ^32^, ^33^ However, the effect of aberrant *SEPT9* hypermethylation on gene mRNA expression is inconsistent across different studies.^32^, ^34^, ^35^

In this study, cg14517217 showed hypermethylation in the tumor tissue of lung adenocarcinoma, which was significantly correlated with *SEPT9* expression. The biological function studies indicated that mRNA and protein expression of *SEPT9* were downregulated in lung adenocarcinoma cell lines after dealing with 5‐aza‐2dC in different concentration gradients. This suggests that cg14517217 may have a certain effect on the occurrence and development of lung adenocarcinoma through positively regulating the expression of *SEPT9*. A similar trend was seen in cg15386964, which may play a vital role in lung adenocarcinoma by upregulating the expression of *HIST1H2BH*.^36^, ^37^, ^38^ In contrast, cg18878992 was found to be negatively correlated with *MAPT* mRNA expression, showing that cg18878992 may influence the development of lung adenocarcinoma by downregulating the expression of *MAPT* ^39^, ^40^, ^41^. The functional relationship between these three methylated CpG sites and lung adenocarcinoma remains to be explored.

In this study, systematic exploration of methylated CpG sites was performed to predict lung adenocarcinoma prognosis through TCGA analysis, and the results obtained from the Chinese population and biological function studies provide effective evidence that DNA methylation biomarker can affect the process of occurrence and development of lung adenocarcinoma. As this methylation signature can be performed to evaluate the prognosis both in TCGA and Chinese lung adenocarcinoma, suggesting that this model may provide new genetic factors of lung adenocarcinoma and can help to prognostic predictions in the clinic. However, there are still some limitations in this study. First, only DNA methylation effect was considered for lung adenocarcinoma prognosis study. The development of lung adenocarcinoma may be related to other types of variants. Other types of variants included in subsequent studies will be helpful to know more about the effect of DNA methylation in cancer. Second, the population for validation with Chinese cohort was not large enough. The sample size needs to be increased for further research in order to verify the role of DNA methylation in lung adenocarcinoma.

## 5 CONCLUSIONS

In conclusion, this is the first study to employ a three‐CpG‐based model for lung adenocarcinoma, which is a feasible and useful prognostic method in prognostic prediction which was validated in a Chinese cohort. Additional Chinese population studies with larger sizes and biological function studies in animal models are necessary for our finding verification.

## CONFLICT OF INTEREST

The authors have no conflict of interest.

## ETHICS APPROVAL

This project was approved by the Institutional Review Board of Huazhong University of Science and Technology.

## Supporting information

Supplementary Material

## Data Availability

I confirm that the data collected during and analyzed in this study are available from the corresponding author upon reasonable request.
