# Supplementary material for: A functional methylation signature to predict the prognosis of Chinese lung adenocarcinoma based on TCGA
Source: Cancer Med. 2021 Dec 2;11(1):281–94. doi: 10.1002/cam4.4431 (PMC8704183; doi:10.1002/cam4.4431)

Supplementary Table 1 Gene annotation information of three methylation CpG islands selected by LASSO COX regression in Human Methylation450 array

**Methylation**	**Gene symbol**	**Gene type**	**Position to TSS (bp)**	**CGI Coordinate**	**Feature Type**
cg14517217	*SEPT9*	protein coding	78205; 658; 819; 151	chr17:77451395-77451739	Island
cg15386964	*HIST1H2BH*	protein coding	-1195	chr6:26250208-26250599	Island
cg15386964	*HIST1H3F*	protein coding	149		Island
cg18878992	*MAPT*	protein coding	2595; 2303	chr17:45894044-45897674	Island
cg18878992	*MAPT-AS1*	antisense	-1377; -1464		Island

Supplementary Table 2 LASSO COX coefficients and fold change of three methylation CpG islands associated with the prognosis of lung adenocarcinoma in TCGA set

Methylaion CpGs	LASSO COX coefficients	Fold change （tumor vs. adjacent）
cg14517217	0.45	6.84
cg15386964	0.04	8.46
cg18878992	-0.17	5.92

Supplementary Table 3 The identified CpG islands methylation levels grouped by clinical and pathological characteristics of lung adenocarcinoma patients in TCGA

Characteristics	N	cg14517217	cg15386964	cg18878992	Methylation signature
Age (years)					
<65	196	0.17 ± 0.22	0.28 ± 0.25	0.18 ± 0.18	0.04 ± 0.10
≥65	222	0.14 ± 0.20	0.33 ± 0.24	0.16 ± 0.16	0.05 ± 0.09
P value		0.158	0.036	0.278	0.444
Sex					
Male	195	0.15 ± 0.20	0.32 ± 0.25	0.17 ± 0.17	0.05 ± 0.10
Female	223	0.14 ± 0.22	0.31 ± 0.24	0.18 ± 0.17	0.04 ± 0.10
P value		0.921	0.971	0.532	0.717
Race					
White	336	0.15 ± 0.21	0.32 ± 0.24	0.17 ± 0.17	0.05 ± 0.10
Others	82	0.14 ± 0.20	0.27 ± 0.26	0.18 ± 0.18	0.04 ± 0.10
P value		0.858	0.109	0.982	0.767
Smoking status					
Yes	339	0.16 ± 0.22	0.32 ± 0.25	0.18 ± 0.18	0.06 ± 0.10
No	61	0.08 ± 0.14	0.29 ± 0.24	0.15 ± 0.15	0.02 ± 0.07
P value		0.003	0.446	0.232	0.010
Chemotherapy history					
Yes	152	0.16 ± 0.22	0.33 ± 0.24	0.16 ± 0.16	0.06 ± 0.10
No	266	0.15 ± 0.20	0.30 ± 0.25	0.18 ± 0.18	0.05 ± 0.09
P value		0.407	0.297	0.102	0.189
Radiotherapy history					
Yes	88	0.14 ± 0.20	0.28 ± 0.23	0.15 ± 0.16	0.05 ± 0.09
No	330	0.15 ± 0.21	0.32 ± 0.25	0.18 ± 0.17	0.05 ± 0.10
P value		0.697	0.207	0.085	0.949
Clinical stage					
Stage I	227	0.13 ± 0.19	0.29 ± 0.23	0.18 ± 0.17	0.04 ± 0.09
Stage II/III/IV	186	0.18 ± 0.23	0.34 ± 0.26	0.17 ± 0.17	0.07 ± 0.11
P value		0.013	0.046	0.658	0.007

Supplementary Table 4 Baseline characteristics of lung adenocarcinoma patients in Chinese lung adenocarcinoma patients (n=116)

Characteristic	Results
Age (years)	60.27 ± 8.57
Sex	
Male	56 (48.3%)
Female	60 (51.7%)
Smoking status	
Yes	45 (38.8%)
No	71 (61.2%)
Clinical stage	
Stage I	39 (33.6%)
Stage II	30 (25.9%)
Stage III	42 (36.2%)
Stage IV	5 (4.3%)
Lymphatic metastasis	
Yes	61 (52.6%)
No	55 (47.4%)
Differentiation	
No	28 (24.1%)
Low	13 (11.2%)
Moderate	50 (43.1%)
High	25 (21.6%)
Chemotherapy history	
Yes	40 (34.5%)
No	76 (65.5%)
Radiotherapy history	
Yes	3 (2.6%)
No	113 (97.4%)
Dead	
Yes	20 (17.2%)
No	96 (82.8%)
Survival time (month)	24.18 (16.71-27.00)

Supplementary Table 5 Amplification primers and sequencing primers for the three methylation CpG islands

**Methylation**	**RefGene**	**Sequence**	**Primer sequence**
cg14517217	*SEPT9*	Forward primer	GTGGTGTAAGGTTGGGAAGAATAAA
		Reverse primer	ACCTCCCTTCCCAAACAATC
		Sequencing	GATTAAAGGGGTTTTGTG
cg15386964	*HIST1H2BH*	Forward primer	GGAATGGTAGTTTGAGAATTAGTAGTTTAG
		Reverse primer	ACCATTACTTAAAACCCATTCCTATAAC
		Sequencing	AGTAGTTTAGTAGATTTTTGATAG
cg18878992	*MAPT*	Forward primer	GGAAGAGTATTTAGGAGTTGAAT
		Reverse primer	ATACCCAAACTCCAACCCTCTA
		Sequencing	GAGTATTTAGGAGTTGAATTTTTTA

Supplementary Table 6 The identified CpG islands methylation levels grouped by clinical and pathological characteristics of Chinese lung adenocarcinoma patients

**Characteristics**	**N**	**cg14517217**	**cg15386964**	**cg18878992**	**Methylation signature**
Age (years)					
<65	55	0.02 ± 0.03	0.13 ± 0.16	0.09 ± 0.12	-0.03 ± 0.26
≥65	61	0.04 ± 0.09	0.16 ± 0.15	0.07 ± 0.06	0.12 ± 0.43
P value		0.083	0.292	0.153	0.021
Sex					
Male	56	0.04 ± 0.08	0.15 ± 0.16	0.07 ± 0.08	0.11 ± 0.42
Female	60	0.02 ± 0.05	0.14 ± 0.16	0.08 ± 0.10	-0.01 ± 0.29
P value		0.054	0.735	0.783	0.065
Smoking status					
Yes	45	0.05 ± 0.09	0.15 ± 0.15	0.09 ± 0.11	0.15 ± 0.46
No	71	0.02 ± 0.05	0.14 ± 0.16	0.08 ± 0.07	-0.02 ± 0.28
P value		0.011	0.790	0.614	0.013
Chemotherapy history					
Yes	40	0.03 ± 0.06	0.16 ± 0.19	0.09 ± 0.10	0.06 ± 0.38
No	76	0.02 ± 0.07	0.14 ± 0.13	0.08 ± 0.09	0.04 ± 0.35
P value		0.972	0.449	0.391	0.786
Radiotherapy history					
Yes	3	0.01 ± 0.00	0.28 ± 0.26	0.07 ± 0.09	0.05 ± 0.37
No	113	0.03 ± 0.07	0.14 ± 0.15	0.08 ± 0.09	0.01 ± 0.07
P value		0.389	0.155	0.505	0.801
Clinical stage					
Stage I	39	0.02 ± 0.07	0.11 ± 0.14	0.06 ± 0.09	0.04 ± 0.36
Stage II/III/IV	77	0.03 ± 0.07	0.17 ± 0.16	0.09 ± 0.09	0.05 ± 0.37
P value		0.708	0.052	0.196	0.025

Supplementary Table 7 Amplification primers of the three genes for PCR

Gene	Primer	Primer sequence	Size
GAPDH	Forward primer	5‘- TCAAGAAGGTGGTGAAGCAGG -3’	115bp
	Reverse primer	5‘- TCAAAGGTGGAGGAGTGGGT -3’	
SEPT9	Forward primer	5‘- GGGCGTGAAGAACTCAGAAC -3’	157bp
	Reverse primer	5‘- CTGCTTGGACGAGATGTCAA -3’	
HIST1H2BH	Forward primer	5‘- AGAAGGCGGTGACCAAGGC -3’	112bp
	Reverse primer	5‘- GTGTCGGGGTGGACTTGCTT -3’	
MAPT	Forward primer	5‘- TAGCAACGTCCAGTCCAAGT -3’	219bp
	Reverse primer	5‘- AGGGACCCAATCTTCGACTG -3’	

Supplementary Figure legends

Supplementary Figure 1 The methylation level and gene expression level of three methylation CpG islands associated with the prognosis of lung adenocarcinoma among tumor and adjacent non-tumor tissues in TCGA set.
(A) cg14517217 and *SEPT9*. **(B)** cg15386964 and *HIST1H2BH*. **(C)** cg18878992 and *MAPT*.

Supplementary Figure 2 Pyrosequencing of three methylation CpG islands was used to validate the result of Illumina Methylation 450K Beadchip assay in TCGA set.
(A) cg14517217. **(B)** cg14517217. **(C)** cg15386964.

Supplementary Figure 3 The methylation level and gene expression level of three methylation CpG islands associated with the prognosis of lung adenocarcinoma among tumor and adjacent non-tumor tissues in Chinese people.
(A) cg14517217 and *SEPT9*. **(B)** cg15386964 and *HIST1H2BH*. **(C)** cg18878992 and *MAPT*.

Supplementary Figure 4 The protein expression of three methylation CpG islands in three cell lines by Western blotting.
(A) Western blotting showing the protein expression of three methylation CpG islands in cells demethylated with 5-aza-2dC in different concentration. **(B)** The protein expression of three methylation CpG sites in cells demethylated with 5-aza-2dC.
** P<0.001.

Supplementary Figure 1

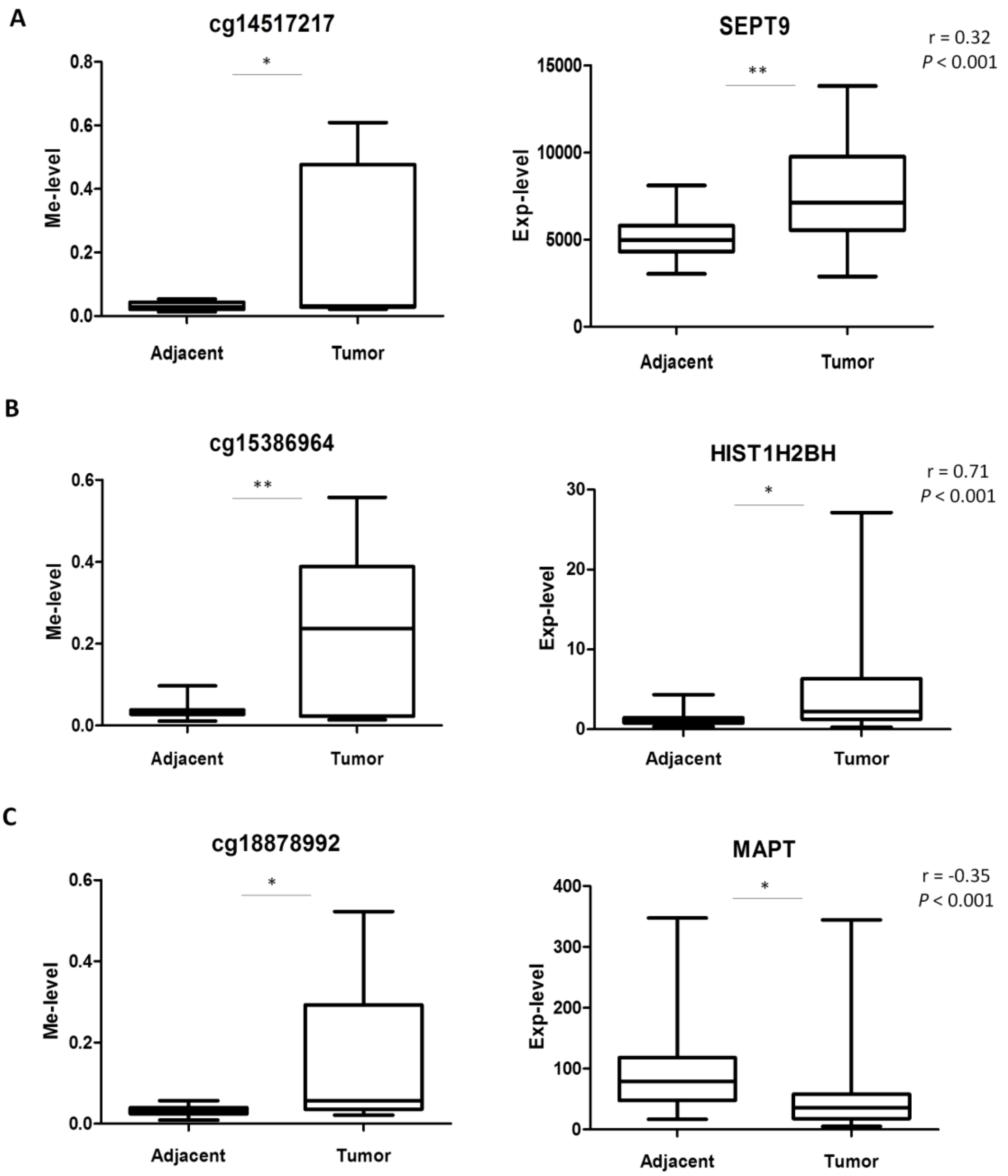

Supplementary Figure 2

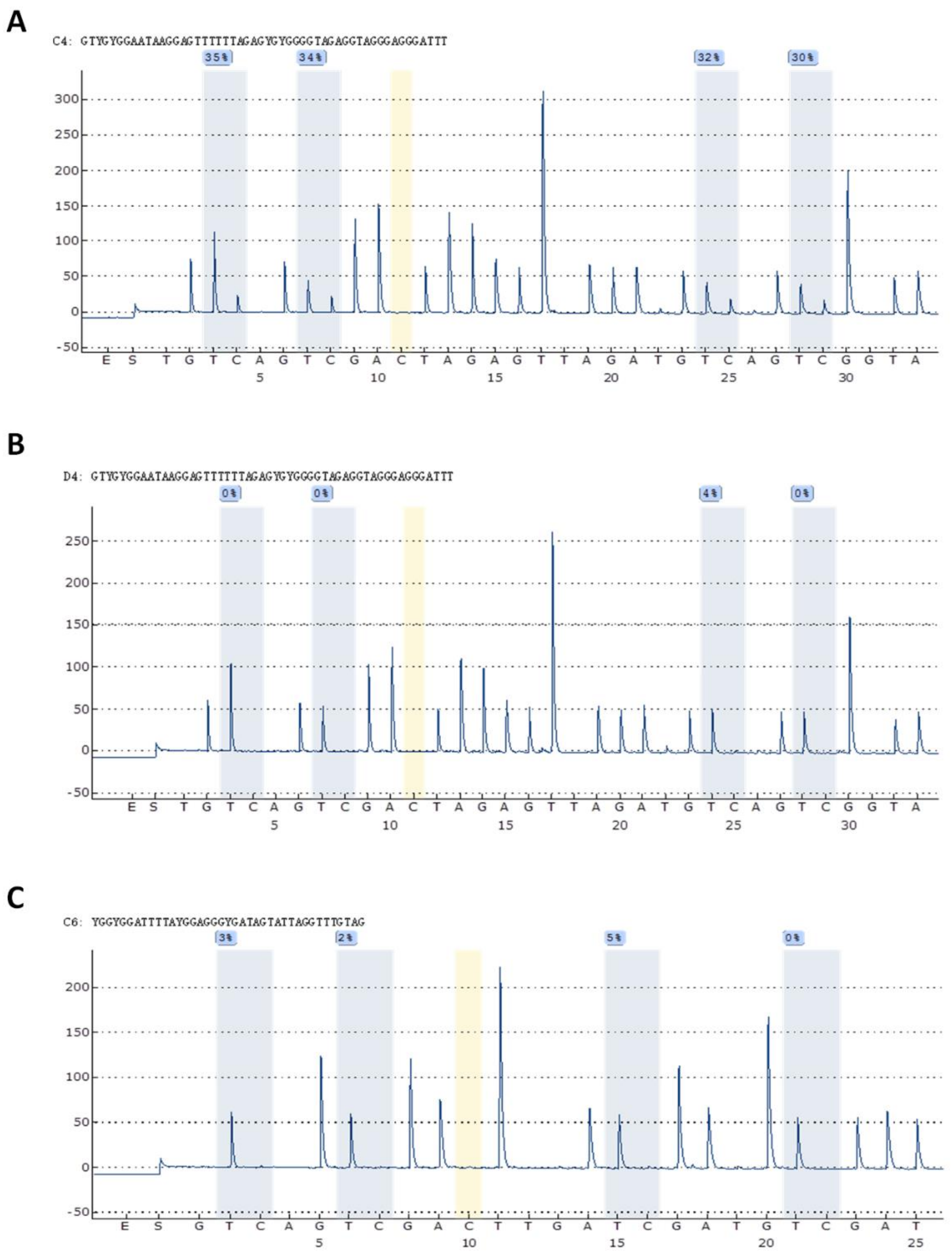

Supplementary Figure 3

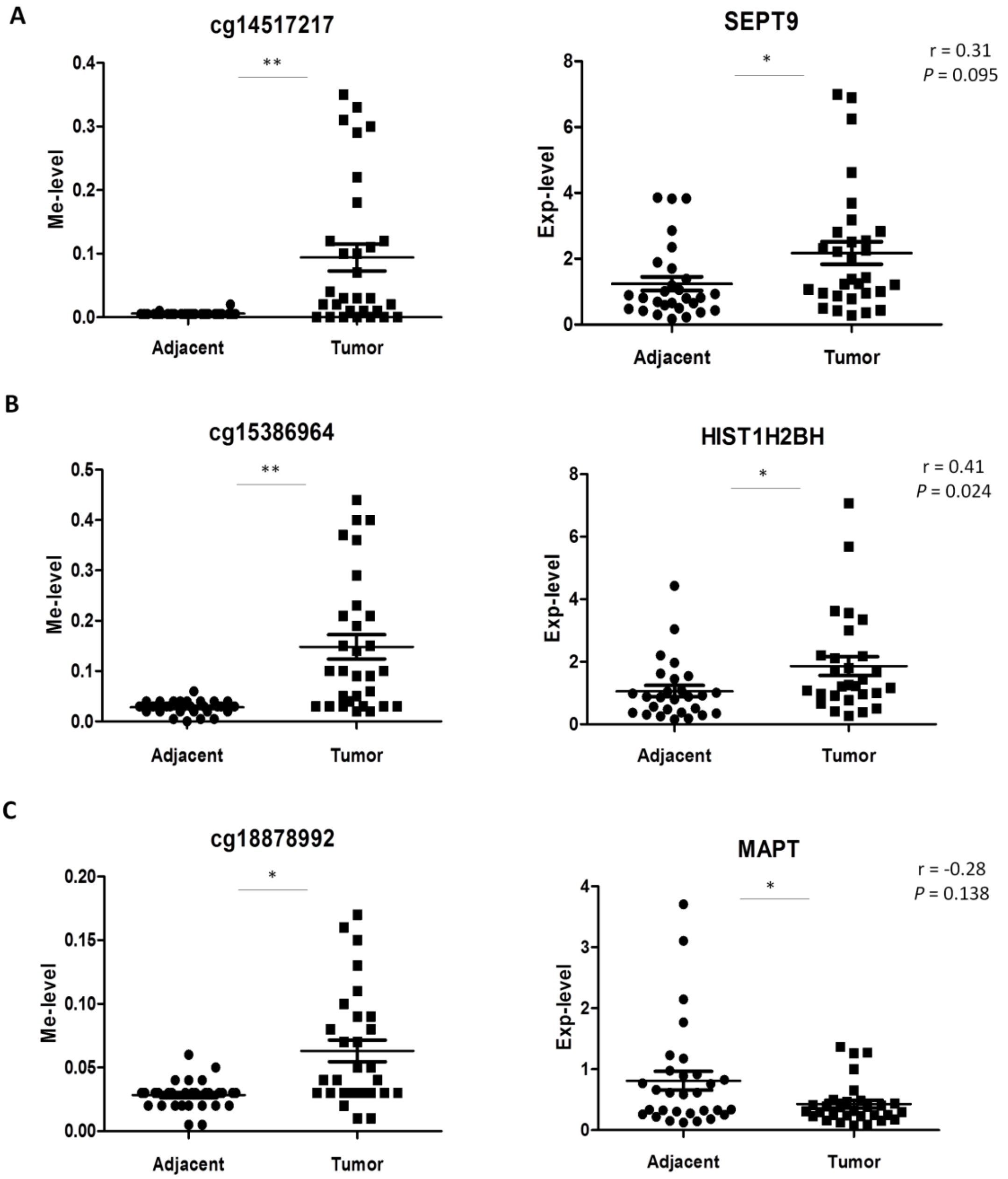

Supplementary Figure 4

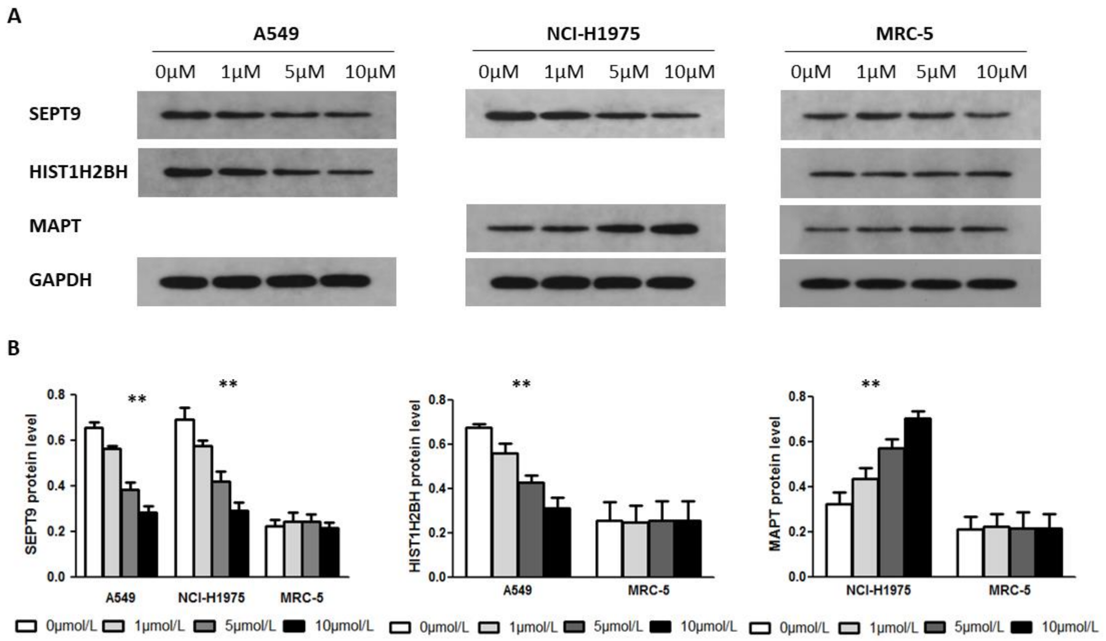

Supplement: Supplementary file 1 — Supplementary Material [file CAM4-11-281-s001.pdf]
